# Apgar Scoring System in Brazil's Live Births Records: Differences between Home and Hospital Births

**DOI:** 10.1055/s-0038-1675572

**Published:** 2018-12-12

**Authors:** Jordana de Faria Bessa, Naieli Bonatto

**Affiliations:** 1 Department of Gynecology, Clínica Humana, Guarulhos, SP, Brazil; 2 Department of Radiology and Oncology, Universidade de São Paulo, São Paulo, SP, Brazil

**Keywords:** home birth, normal birth, apgar score, parto domiciliar, parto normal, escala apgar

## Abstract

**Objective** To promote informed choice for women and to compare home and hospital births in relation to the Apgar score.

**Methods** Mother's profile and Apgar score of naturally born infants (without forceps assistance) in Brazil between 2011 and 2015, in both settings—hospital or home—were collected from live birth records provided by the Informatics Department of the Unified Health System (DATASUS, in the Portuguese acronym). For the analysis, were included only data from low-risk deliveries, including gestational time between 37 and 41 weeks, singleton pregnancy, at least four visits of prenatal care, infants weighing between 2,500 g, and 4,000 g, mother age between 20-40 years old, and absence of congenital anomalies.

**Results** Home birth infants presented significantly higher risk of 0-5 Apgar scores, both in 1 minute (6.4% versus 3%, odds ratio [OR] = 2.2, confidence interval [CI] IC 2–2.4) and in 5 minutes (4.8% versus 0.4%, OR = 11.5, CI 10.5–12.7). Another finding is related to recovery estimates when from an initially bad 1-minute Apgar (< 6) to a subsequently better 5-minute Apgar (> 6). In this scenario, home infants had poorer recovery, Apgar score was persistently < 6 throughout the fifth minute in most cases (71% versus 10.7%, OR 20.4, CI 17–24.6).

**Conclusion** The results show worse Apgar scores for babies born at home, compared with those born at the hospital setting. This is a pioneer and preliminary study that brings attention concerning differences in Apgar score related to home versus hospital place of birth in Brazil.

## Abstract (pt)

**Resumo:**

**Objetivo** Promover a escolha informada para as mulheres, comparando os resultados de partos domiciliares e hospitalares em relação à escala de Apgar.

**Métodos** Foram coletadas as informações maternas e a pontuação Apgar de nascidos de parto normal (pela definição, sem auxílio de fórcipe) no Brasil, de 2011 a 2015, a partir de registros de nascidos vivos disponibilizados pela plataforma do Departamento de Informática do Sistema Único de Saúde (DATASUS). Para a análise, incluímos somente dados de partos de baixo risco ocorridos em hospitais ou residências, incluindo tempo de gestação entre 37 e 41 semanas, gestação única, pelo menos quatro consultas de pré-natal, crianças com peso entre 2.500 g e 4.000 g, e idade materna entre 20 anos e 40 anos e ausência de anomalias congênitas.

**Resultados** Em comparação ao nascido em ambiente hospitalar, o nascido em domicílio apresentou risco significativamente maior de pontuação 0 a 5, tanto no primeiro minuto (6,4% versus 3%, razão de chance [RC] = 2,2, intervalo de confiança [IC] 2-2,4) como no quinto minuto (4,8% versus 0,4%; RC = 11,5; IC 10,5–12,7). Outro achado que merece destaque é em relação às estimativas de recuperação quando de um Apgar inicialmente ruim ao primeiro minuto (< 6) para um subsequente melhor (> 6) no quinto minuto. Neste cenário, os nascidos em domicílio apresentaram menor recuperação até o quinto minuto, persistindo em Apgar < 6 na maior parte dos casos (71% versus 10,7%; OR 20,4; IC 17-24,6).

**Conclusão** Os resultados indicam piores escalas de Apgar para bebês nascidos em ambiente domiciliar, em comparação àqueles nascidos em ambiente hospitalar. Este é um estudo pioneiro e preliminar que atenta para as diferenças na escala de Apgar em relação ao local de nascimento domiciliar versus hospitalar no Brasil.

## Introduction

Over the last decades, the number of births by cesarean section has been growing significantly in Brazil, which ranks the country among those carrying out this procedure the most in the world.^1^ ^2^ ^3^ On the other hand, an intense debate has been noticed in the search for the decrease of cesarean section births and the return to home births, which used to be the rule decades ago when the access to hospital health services was not an option.

Around the 1980s, non-governmental organizations and popular fronts started to give this cause a voice. Designations such as “obstetric violence” against “humanized birth” became popular, driven by growing reports of abuse from birth assistance in public and private hospitals. Those movements intend to recover the mother's autonomy and her main role in the process of giving birth.^4^ ^5^

The year of 1993 was the milestone for the fight against the so called “obstetric violence,” with the foundation of the Network for the Humanization of Labor and Birth (ReHuNa, in the Portuguese acronym)*,* a non-profit organization that offers help reporting violence and embarrassing circumstances, which can turn the birth experience into one of “terror, anguish, helplessness, alienation and pain.”^6^

In 2011, the United Nations (UN) Convention on the Elimination of All Forms of Discrimination against Women Committee sentenced the State of Brazil to pay compensation for the maternal death of a 28-year-old woman deceased in 2002, victim of medical assistance negligence during gestation.^7^

The matter regained repercussion in March 2014, when attorneys representing a hospital filed a petition with the Court to interrupt a home birth. According to the report, the measure was justified because the parturient had undergone two previous cesarean sections and the fetus was in breech position. The woman refused to undergo cesarean section and left the hospital. The petition was successful, and the parturient was coerced to return. She then received manifestations of support from several national and international authorities, including the Federal Government's Office of Human Rights.^8^

Some of the most frequent reported reasons for choosing home births are: fewer interventions, sensation of being in control, a comfortable environment, and bad previous experiences in the hospital.^9^ The choice for home birth is also made when the mother does not agree with the recommendation of a cesarean section given in the hospital, such as in cases of breech, twin pregnancy and previous cesarean sections.^10^

The Health Secretary, through the “*Rede Cegonha*” program,^11^ requires from public governments the compliance of the World Health Organization's (WHO) 1996 document “Care in Normal Birth: A Practical Guide,”^12^ which includes the respect of mother's choice of birthplace among other recommended measures.

No study comparing neonatal results of home and hospital births was ever conducted in Brazil. Apgar scoring is a worldwide recognized system that remains to this day as an important neonatal prognosis tool as it was described 65 years ago.^13^ The purpose of this study is to promote informed choice for women and provide information on safety of place of birth (hospital births compared with home births) in Brazil based on 1 and 5-minute Apgar score distribution. Although the Apgar score alone does not predict long-term outcomes, its importance can be assured by the fact that infants scoring 0 to 3 in 5-minute Apgar score are related to increased mortality in the first week of life.^14^ This is a pioneer study conducted in Brazil whose results can be useful for future investigations and to help planning strategies on childbirth care.

## Methods

This study comprises population retrospective analysis based on live births records provided by the Informatics Department of the Unified Health System (DATASUS, in the Portuguese acronym) in cooperation with the Live Birth Information System (SINASC, in the Portuguese acronym).^15^ These are online public access data platform administered by Brazilian government that includes the records of births throughout Brazilian territory, collected from health care intuitions or notary offices (for home births). DATASUS-SISNAC provides a rich source of data, mostly unexploited yet by the scientific community. Despite of some missing data, for example, individual data, still births and, in case of hospital births, if it was initially planned at home and then rushed to the hospital, this is the most complete source of information for births in Brazil.

**Fig. 1:**
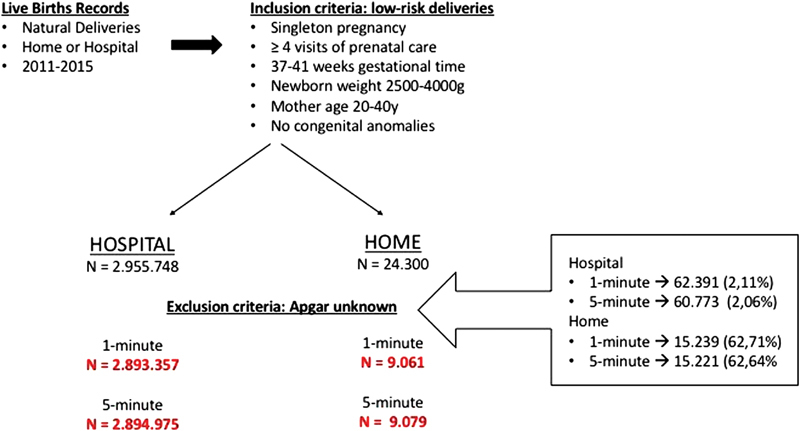
Study design.

Mother profile and Apgar score of naturally born infants (without forceps assistance) in Brazil, between 2011 and 2015, in both settings—home and hospital—were collected. To avoid possible bias among comparisons due to risk childbirth, a choice was made to include only deliveries considered to be of good prognosis, from low-risk pregnancies, including gestational time between 37 and 41 weeks, singleton pregnancy, with at least four visits of prenatal care, infants weighing between 2,500-4,000 g, mother age between 20-40 years old, and absence of congenital anomalies. Schematic representation for methodology is presented in Fig. 1.

Apgar scoring system was set as endpoint because of its worldwide recognition tool as an important neonatal prognosis marker, as described by Dr. Virginia Apgar in 1953.^13^ The scoring system ranges from zero to ten and corresponds to the summing up of the score obtained by the evaluation of five criteria: heart rate, respiration, muscle tone, reflex irritability and color. Distribution of newborns was obtained according to different 1-minute and 5- minute Apgar zones: 0–2, 3–5, 6–7, 8–10. These zones are the usual presentation at the birth certificate. Thus, birth records without Apgar score were excluded. Three ratios were then obtained: 1) the proportion of newborns presenting with the bottom scores, 0–2 and 3–5, in relation to birthplace, at the first minute; 2) the same, at the fifth minute; 3) newborns who, presenting low 1-minute scores, sustained 5-minute scores below 6, expressing poor recovery (it is unknown whether they did recover at 10, 15 or 20 minutes, because this information is not usually provided at the birth certificate). Chi-square, OR (odds ratio), and Fisher exact tests were calculated with GraphPad Prism software, version 7 (GraphPad Software Inc., La Jolla, CA, USA) and were considered significant if *p* < 0.05.

## Results

The cohort included 24,300 newborns born at home, and 2,955,748 born at the hospital, as shown in Table 1. Some characteristics were associated with a higher rate of home births: North region (35/1,000); indigenous ethnicity (201/1,000); no schooling (75/1,000) or less than 4 years (25/1,000).

**Table 1:** Characteristics of live birth population from natural birth, according to birthplace

Total	**Hospital**	**Home**
	2,955,748 (99.18%)	24,300 (0.82%)
Region		
North	294,545 (96.50%)	10,673 (3.50%)
Northeast	846,091 (99.31%)	5,867 (0.69%)
Southeast	1,207,120 (99.58%)	5,097 (0.42%)
South	400,799 (99.69%)	1,238 (0.31%)
Central-West	207,193 (99.32%)	1,425 (0.68%)
Mother's age		
20–24 years	1,186,664 (99.29%)	8,443 (0.71%)
25–29 years	925,778 (99.20%)	7,435 (0.80%)
30–34 years	589,634 (99.04%)	5,691 (0.96%)
35–39 years	253,672 (98.93%)	2,731 (1.07%)
Ethnicity		
White	968,838 (99.48%)	5,049 (0.52%)
Black	168,745 (99.28%)	1,224 (0.72%)
Asian	11,501 (99.16%)	97 (0.84%)
Mixed	1,698,338 (99.27%)	12,441 (0.73)
Indigenous	18,988 (79.36%)	4,938 (20.64%)
Ignored	89,338 (99.39%)	551 (0.61%)
Marital status		
Single	1,296,900 (99.31%)	9,067 (0.69%)
Married	849,893 (99.35%)	5,579 (0.65%)
Widowed	5,683 (99.39%)	35 (0.61%)
Divorced	28,366 (99.44%)	161 (0.56%)
Consensual union	744,315 (98.80%)	9,009 (1.20%)
Ignored	30,591 (98.55%)	449 (1.45%)
Schooling		
None	22,220 (92.49%)	1,803 (7.51%)
1–3 years	139,994 (97.47%)	3,641 (2.53%)
4–7 years	678,532 (98.88%)	7,710 (1.12%)
8–11 years	1,783,070 (99.63%)	6,707 (0.37%)
≥ 12 years	291,301 (98.78%)	3,611 (1.22%)
Ignored	40,631 (98.00%)	828 (1.20%)

1-minute Apgar: 62.7% of home births did not have 1-minute Apgar scores registered at the birth certificate, in contrast to 2.1% of hospital births. These cases were excluded. The newborns distribution according to different 1-minute Apgar scores are shown in Table 2. As noted, 6.4% of newborns from home births and 3% from hospital births had 1-minute Apgar scores below 6; therefore, the chance of a low score was 2.2 times higher for home births [OR = 2.2 (IC 2–2.4); *p* < 0.0001].

**Table 2:** Distribution of newborns according to different 1-minute Apgar score by birthplace

Birthplace	Hospital		Home	
	n	%	n	%
Apgar < 6	86,179	2.98%	577	6.37%
0–2	16,325	0.56%	418	4.61%
3–5	69,854	2.41%	159	1.75%
Apgar ≥ 6	2,807,178	97.02%	8,484	93.63%
6–7	244,837	8.46%	1,009	11.14%
8–10	2,562,341	88.56%	7,475	82.50%
Total	2,893,357	100%	9,061	100%

5-minute Apgar: a high rate of uninformed Apgar on the records was also seen, 62.6% of home and 2.1% of hospital births. These cases were excluded for this analysis. The newborns distribution according to different 5-minute Apgar are shown in Table 3. Apgar scores below 6 were seen in 4.8% of newborns at home, and 0.4% of newborns at the hospital. For 5-minute Apgar, the chance of a low score was 11.5 higher for home compared with hospital births (OR = 11.5[IC 10.5-12.7]; *p* < 0.0001).

**Table 3:** Distribution of newborns according to different 5-minute Apgar score by birthplace

Birthplace	Hospital		Home	
	n	%	n	%
**Apgar < 6**	12,718	0.44%	439	4.84%
0–2	4,526	0.16%	387	4.26%
3–5	8,192	0.28%	52	0.57%
**Apgar ≥ 6**	2,882,257	99.56%	8,640	95.16%
6–7	38,100	1.32%	107	1.18%
8–10	2,844,157	98.24%	8,533	93.99%
**Total**	2,894,975	100%	9,079	100%

Persistence of Apgar < 6 from 1- to 5-minute: Persistence in low score means both 1-minute and 5-minute below 6. Likewise, recovery was defined as a 5-minute Apgar above 6 succeeding a 1-minute Apgar below 6. The analysis was not possible when 1-minute score was low, but the 5-minute score was not informed (0.2% of hospitals and 4.3% of home records were excluded). The results are shown in Table 4. The chance of a persistent low Apgar score was 20.4 times higher for home compared with hospital newborns (OR = 20.4 [IC 17-24.6]; *p* < 0.0001). When presenting a low 1-minute Apgar score, recovery to higher values was seen in almost 90% of the newborns at the hospital and only 29% of those at home.

**Table 4:** Persistence of Apgar < 6, from 1-minute to 5-minute, by birthplace

Birthplace	Hospital		Home	
	n	%	n	%
Apgar < 6	9,213	10.71%	392	71.01%
0–2	1,814	2.11%	371	67.21%
3–5	7,399	8.60%	21	3.80%
Apgar ≥ 6	76,840	89.29%	160	28.99%
6–7	27,349	31.78%	33	5.98%
8–10	49,491	57.51%	127	23.01%
Total	86,053	100%	552	100%

Although 20% of indigenous newborns were delivered at home, ethnicity was not accountable for the differences seen above. Among total indigenous newborns, 2.94% had Apgar score below 6, similar to the rate found for hospital deliveries. It is worth notice that the North region has one of the lowest ratios of newborns with low Apgar scores (2.13%). Some characteristics were associated with higher ratios, including: Northeast Region (3.05%), Southeast region (3.32%); mother age 30–34 years old (3.06%), mother age 35–40 years old (3.43%); white (3.20%), black (3.19%) and Asian ethnicity (3.53%); married (3.24%); schooling 8-11 years (3.06%) and schooling ≥12 years (3.60%). More details about the distribution of newborns by Apgar score zone and mother profile can be seen in Appendix 1 and 2.

**Appendix 1:** Newborn distribution by Apgar score zones, birthplace, and mother profile: first minute

Score zones	0–2	3–5	6–7	8–10	Total	Apgar < 6 (%)	DP (MIN)	DP (MAX)
Birthplace								
Hospital	**16,325**	**69,854**	**244,837**	**2,562,341**	**2,893,357**	**2,978512503**	**3,00**	**2,96**
Home	418	159	1,009	7,475	9,061	6.37	6,89	5,88
Region								
North	1,222	5,015	31,052	255,767	293,056	2.13	2,18	2,08
Northeast	4,268	20,368	84,996	699,416	809,048	3.05	3,08	3,01
Southeast	7,860	31,722	85,391	1,068,795	1,193,768	3.32	3,35	3,28
South	2,458	9,036	28,842	359,634	399,970	2.87	2,93	2,82
Central-West	935	3,872	15,565	186,204	206,576	2.33	2,39	2,26
Mother's age (years)								
20–24	6,228	27,522	101,832	1,027,136	1,162,718	2.90	2,93	2,87
25–29	5,159	21,497	75,590	806,489	908,735	2.93	2,97	2,90
30–34	3,546	14,236	47,743	515,317	580,842	3.06	3,11	3,02
35–39	1,810	6,758	20,681	220,874	250,123	3.43	3,50	3,35
Ethnicity								
White	6,079	24,773	73,893	858,056	962,801	3.20	3,24	3,17
Black	1,047	4,217	13,257	146,493	165,014	3.19	3,28	3,11
Asian	70	330	845	10,101	11,346	3.53	3,88	3,20
Mixed	8,931	38,208	149,009	1,462,282	1,658,430	2.84	2,87	2,82
Indigenous	118	459	1,801	17,243	19,621	2.94	3,19	2,71
Ignored	498	2,026	7,041	75,641	85,206	2.96	3,08	2,85
Marital status								
Single	7,381	30,305	104,601	1,130,868	1,273,155	2.96	2,99	2,93
Married	5,206	21,954	71,657	738,610	837,427	3.24	3,28	3,21
Widowed	32	123	460	4,933	5,548	2.79	3,26	2,39
Divorced	168	647	1,992	25,303	28,110	2.90	3,10	2,71
Consensual union	3,809	16,252	64,218	645,189	729,468	2.75	2,79	2,71
Ignored	147	732	2,918	24,913	28,710	3.06	3,27	2,87
Schooling (years)								
None	132	525	2,326	18,708	21,691	3.03	3,27	2,81
1–3	817	2,854	13,093	118,686	135,450	2.71	2,80	2,63
4–7	3,504	13,666	56,086	589,250	662,506	2.59	2,63	2,55
8–11	10,129	43,504	146,167	1,553,664	1,753,464	3.06	3,08	3,03
≥ 12	1,943	8,545	24,198	256,735	291,421	3.60	3,67	3,53
Ignored	218	919	3,976	32,773	37,886	3.00	3,18	2,83
Total	16,743	70,013	245,846	2,569,816	2,902,418	2.99	3,01	2,97

**Appendix 2:** Newborn distribution by Apgar score zones, birthplace, and mother profile: fifth minute

Score zones	0–2	3–5	6–7	8–10	Total	Apgar < 6 (%)	DP (MIN)	DP (MAX)
Birthplace								
Hospital	**4,526**	**8,192**	**38,100**	**2,844,157**	**2,894,975**	**0.44**	**0,45**	**0,43**
Home	387	52	107	8,533	9,079	4.84	5,30	4,41
Region								
North	537	703	3,550	287,972	292,762	0.42	0,45	0,40
Northeast	1,611	2,741	13,546	790,872	808,770	0.54	0,55	0,52
Southeast	2,070	3,324	14,459	1,175,852	1,195,705	0.45	0,46	0,44
South	384	955	4,536	394,298	400,173	0.33	0,35	0,32
Central-West	311	521	2,116	203,696	206,644	0.40	0,43	0,38
Mother's age (years)								
20–24	1,865	3,131	15,171	1,142,907	1,163,074	0.43	0,44	0,42
25–29	1,597	2,475	11,767	893,421	909,260	0.45	0,46	0,43
30–34	982	1,744	7,709	570,877	581,312	0.47	0,49	0,45
35–39	469	894	3,560	245,485	250,408	0.54	0,57	0,52
Ethnicity								
White	1,264	2,691	11,980	947,550	963,485	0.41	0,42	0,40
Black	328	479	2,250	162,225	165,282	0.49	0,52	0,46
Asian	15	29	147	11,162	11,353	0.39	0,52	0,29
Mixed	3,086	4,765	22,552	1,628,757	1,659,160	0.47	0,48	0,46
Indigenous	73	45	200	19,285	19,603	0.6	0,72	0,50
Ignored	147	235	1,078	83,711	85,171	0.45	0,50	0,41
Marital status								
Single	2,190	3,537	16,481	1,251,869	1,274,077	0.45	0,46	0,44
Married	1,329	2,520	11,252	822,765	837,866	0.46	0,47	0,45
Widowed	9	17	60	5,465	5,551	0.47	0,69	0,32
Divorced	43	88	322	27,688	28,141	0.47	0,55	0,39
Consensual union	1,272	1,992	9,646	716,836	729,746	0.45	0,46	0,43
Ignored	70	90	446	28,067	28,673	0.56	0,65	0,48
Schooling (years)								
None	52	82	383	21,151	21,668	0.62	0,73	0,52
1–3	321	446	1,826	132,791	135,384	0.57	0,61	0,53
4–7	1,302	1,804	8,154	651,718	662,978	0.47	0,49	0,45
8–11	2,743	4,871	23,168	1,723,857	1,754,639	0.43	0,44	0,42
≥ 12	407	899	4,142	286,094	291,542	0.45	0,47	0,42
Ignored	88	142	534	37,079	37,843	0.61	0,69	0,53
Total	4,913	8,244	38,207	2,852,690	2,904,054	0.45	0,46	0,45

## Discussion

This study compared 1- and 5-minute Apgar scores of newborns from home births and from hospital births, as provided on births records in Brazil, between 2011 and 2015. In summary, the results show worse Apgar scores for babies born at home compared with those born at the hospital setting.

There were some surprising data. First, ∼ 63% of home births did not have any Apgar score registered in the birth certificate, even though it has is a specific field for this information. It raises a few questions: Why no importance was given to the Apgar score? Were these babies unassisted? Or was it omitted for some reason? It seems more likely that there would be no reasons to omit a high score, but in fact it is not possible to demonstrate such conclusions.

A second surprising finding regards to persistently low scores until the fifth minute. Previous studies show that when both 1- and 5-minute scores are low (more specifically < 4), there is increased risk of death and cerebral palsy.^16^ Our result showed that those born at home had poorer recovery when the score was low at the first minute, being persistently < 6 until the fifth minute in most cases (71% versus 10.7%, OR 20.4, IC 17–24.6). In other words, the chance of recovery until the fifth minute was only 29%. In hospital births, on the other hand, recovery was seen in 89.3%. Neonatal resuscitation maneuvers are a major factor accountable for this difference. This is expected data since neonatal resuscitation resources (such as aspiration cannula, oxygen, ventilation masks, intubation materials, and adrenaline) are readily available in hospitals. Still, the fact that a fast recovery was seen is less than a third of the infants born at home is worrisome.

It is important to remember that the population selected for analysis did not present high-risk factors for complications, such as: mother age under 20 or over 40 years old, preterm or post-term birth, prenatal care with less than four visits, birth weight under 2,500 g or over 4,000 g, or congenital anomalies. It is quite likely that, if those conditions were included, the differences found between home and hospital births would be even greater. Such analysis was not made due to obvious bias to the detriment of home births.

The American College of Obstetricians and Gynecologists (ACOG)^17^ and the American Academy of Pediatrics (AAP)^18^ state that hospitals and maternities are the safest places for natural birth, regardless of the pregnancy risks. Analysis of US births records have also shown poorer outcomes for home births, including higher risks of a null Apgar score (RR 10.5) and neurological dysfunction (RR 3.8).^19^

On the other hand, British entities, such as the Royal College of Obstetricians and Gynaecologists and the Royal College of Midwives, support natural home birth for low-risk pregnancies.^20^ A meta-analysis sponsored by National Institute for Health found no differences between planned home and planned hospital births, regarding mortality, Apgar, neonatal jaundice, ICU transfer, conversion to cesarean section and puerperal hemorrhage.^21^ The method was “intention-to-treat”: the groups were not divided by birthplace, but according to where deliveries were planned to take place. In case of complications during home births, a fast transfer to hospital setting minimizes differences between the groups. It does not undermine the study, it is just not applicable to Brazil. Our lack of urban planning, and in some cases, of ambulances, often make rapid access to hospitals more difficult.

As a strong point of the present study, we highlight the population magnitude and the objectivity of data in birth certificates, which made the analysis less susceptible to mistakes in selection. For instance, in a referred Canadian study frequently used to accredit home births, parturients were checked in by the obstetric nurses hired to assist the delivery themselves.^22^ This kind of experimental design is not ideal, it is naturally biased on account of the nurses, who are knowledgeable of the objective of study and the hypothesis being tested. Dr. Virginia Apgar herself anticipated potential biases when she stated that “it is strongly advised that an observer, other than the person who delivers the infant, be the one to assign the score.”^23^ Those who deliver the infant are invariably emotionally involved with the births and the families, and thus cannot take an accurate decision on assigning the total score. In home births, it is common to have only one provider. The frequent high rate of Apgar = 10 observed in home births is not reliable, more likely a sign of biased calculation.^24^

However, Apgar scores alone do not predict long-term outcomes and may not be an exact representation of birth conditions. There is also great interobserver variance: for the same newborn, two doctors may not give the same score in 18 to 45% of cases.^25^ An Australian study found that, regardless of the fact that live births Apgar scores had been equivalent, there were significant differences in stillborn rates, favoring hospital in comparison to home births.^26^ Thus, for future studies, we suggest follow-up throughout the first week, including data of intrapartum, neonatal, and infant deaths, and, if possible, follow-up of the first year as well, with attention to seizures and signs of neurological dysfunction.

According to our results, it is inferred that home births in Brazil may not establish equal safety in relation to hospital births, especially regarding neonatal resuscitation. Despite preliminary, and to encompass only 1- and 5-minute Apgar scores, they bring concerns about women, health care providers, and politic makers.

The Medical Board Council of São Paulo (CREMESP, in the Portuguese acronym) states that “childbirth care, including low-risk deliveries, should be done in the hospital setting.” The same ordinance demands that “the physician that assists any home birth must report the occurrence.”^27^

As previously mentioned, the reasons women frequently cited when choosing home birth are “fewer interventions, sensation of being in control and comfortable environment.” This information can provide a few tracks. Hospitals should make efforts to improve the mother experience. They must have a full obstetric team that manages to pay attention to each parturient, especially when ob-gyns are busy performing surgeries and cesarean sections, including midwives and back-up physicians. It is mandatory that the hospital provides anesthesia services when required by the patient. Finally, cesarean sections without solid justifications must be avoided.

## Conclusion

The present study found worse 1- and 5-minute Apgar scores for babies born at home, compared with those born at the hospital setting. When presenting 1-minute score < 6, home infants had poorer recovery, Apgar score was persistently < 6 until the fifth minute in most cases (71%). Although the Apgar score alone does not predict long-term outcomes, it remains to this day an important prognostic marker of neonatal death. This is a preliminary study that brings attention and concerns about the safety and training of professionals that conduct home births. It is certainly premature to speculate that home births are implied with higher mortality rates. However, it means the need of additional investigation to pursue if those lower Apgar scores are indeed related with long-term unfavorable outcomes.
